# Evaluation of toxic effects of several carboxylic acids on bacterial growth by toxicodynamic modelling

**DOI:** 10.1186/1475-2859-10-100

**Published:** 2011-11-25

**Authors:** José Antonio Vázquez, Ana Durán, Isabel Rodríguez-Amado, Miguel Angel Prieto, Diego Rial, Miguel Anxo Murado

**Affiliations:** 1 Grupo de Reciclado e Valorización de Materiais Residuais (REVAL), Instituto de Investigacións Mariñas (CSIC), r/Eduardo Cabello, 6. Vigo-36208, Galicia, Spain

## Abstract

**Background:**

Effects of organic acids on microbial fermentation are commonly tested in investigations about metabolic behaviour of bacteria. However, they typically provide only descriptive information without modelling the influence of acid concentrations on bacterial kinetics.

**Results:**

We developed and applied a mathematical model (secondary model) to capture the toxicological effects of those chemicals on kinetic parameters that define the growth of bacteria in batch cultures. Thus, dose-response kinetics were performed with different bacteria (*Leuconostoc mesenteroides*, *Carnobacterium pisicola*, *Escherichia coli*, *Bacillus subtilis *and *Listonella anguillarum*) exposed at increasing concentrations of individual carboxylic acids (formic, acetic, propionic, butyric and lactic). In all bioassays the acids affected the maximum bacterial load (*X_m_*) and the maximum growth rate (*v_m_*) but only in specific cases the lag phase (λ) was modified. Significance of the parameters was always high and in all fermentations the toxicodynamic equation was statistically consistent and had good predictability. The differences between D and L-lactic acid effects were significant for the growth of *E. coli*, *L. mesenteroides *and *C. piscicola*. In addition, a global parameter (*EC*_50,τ_) was used to compare toxic effects and provided a realistic characterization of antimicrobial agents using a single value.

**Conclusions:**

The effect of several organic acids on the growth of different bacteria was accurately studied and perfectly characterized by a bivariate equation which combines the basis of dose-response theory with microbial growth kinetics (secondary model). The toxicity of carboxylic acids was lower with the increase of the molecular weight of these chemicals.

## Background

The study of the inhibitory capacity of antibacterial chemicals on microbial growth is generally based on point estimates of the effect, even though microbial exposure to them may be associated with complex kinetic profiles [1]. Evaluation of the whole time course of observed effects using a toxicodynamic analysis would be a more interesting approach in characterizing the corresponding bacterial responses [2]. Among antibacterial agents, carboxylic acids are commonly applied as preservative of foods, disinfectant of materials and surfaces, agent for the control of fermentations, extracting solvent of biological compounds and substrates for biopolymer manufacturing [3-5]. Different features as susceptibility, adaptability, tolerance, resistance and survival of several bacteria to those weak acids have been extensively reported [6-9]. Thereby, *Escherichia coli *is one of the most commonly studied bacteria because it is a well-known food-poisoning pathogen [10,11]. *Listonella anguillarum *(also know as *Vibrio anguillarum*) is another bacteria that has attracted scientific interest due to its association with high mortalities in aquaculture [12-14] in which probiotics and organic acid treatments are recommended [15-17]. Consequently, the accurate modelling of the acid effects on the microbial growth is necessary and indispensable to describe and compare the efficiencies of different treatments, optimize their inhibitory properties and dosing strategies as well as develop standardized protocols of application.

The toxicodynamic analysis using dose-response (DR) bioassay is a powerful tool widely used in different experimental contexts [18,19]. Based on this perspective, a bivariate model formed by a logistic equation predicting growth profiles and another sigmoid equation simulating DR tendencies was previously investigated to evaluate the simultaneous effects of detergent [20], alkyl esters [21] and heavy metals [22] on microbial growth or mortality. In this type of models the most representative kinetic parameters (maximum bacterial load, lag phase and maximum growth rate) are non-linearly affected by the concentration or dose of a chemical. Furthermore, simultaneous fits of all experimental data from control and toxic-dosed cultures must be accomplished for correct modelling [22]. This proposal is experimentally more realistic than the conventional toxicological assessment that is focused on the estimation of the specific growth rate from biomass quantified at two data in the exponential phase [23].

The aim of the present work is to evaluate the effect of carboxylic acids on different bacteria using a toxicodynamic model. This mathematical model was formulated by the combination of the Weibull equation for dose-response description and the logistic equation for bacterial growth in a bivariate model. Data from growth curve studies of *Leuconostoc mesenteroides *(Ln), *Carnobacterium pisicola *(Cb), *E. coli *(Ec), *Bacillus subtilis *(Bs) and *L. anguillarum *(La) exposed to a wide range of concentrations of five carboxylic acids (formic, acetic, propionic, butyric and lactic) is used for model validation. The accuracy and suitability of the proposed model is extensively demonstrated in all bacterial kinetics and the numerical estimates allow us a complete toxic characterization of carboxylic acid effects on growth parameters.

## Results

**Figure 1:**
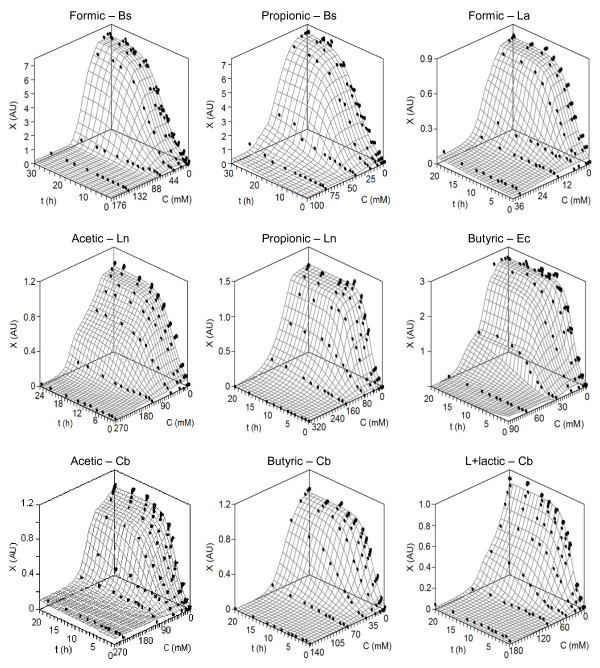
**Experimental data of the growth kinetics for different selected bacteria and acids (points), and fittings to equation (1) (surfaces)**. *X*: growth as optical density at 700 nm (AU); *C*: acid concentration (mM); *t*: time (h). For clarity, confidence intervals (in all cases less than 5% of the experimental mean value; α = 0.05; n = 3) were omitted. Keys for bacteria are described in Table 7.

Selected representation of the tested combinations is depicted in Figure 1 where experimental data from each culture were simultaneously fitted to equation (1). In all cases, surfaces predicted by the model showed good agreement with data obtained in the bioassays. Graphical responses indicated the difference of bacterial sensitivity to the acids exposed. For instance, formic acid was more toxic for La growth than for the production of biomass by Bs. Table 1 summarizes the parametric estimates and statistical analysis of the effects generated by formic and acetic acids on the growth of the five bacteria tested. In most of cultures affected by those acids the effects on lag phase were statistically not significant. Only in the cultures of Cb and Ln dosed with formic, significant effects for *λ*-parameter were observed.

**Table 1:** Parametric estimates and confidence intervals (α = 0.05) corresponding to equation (1) applied to the effect of formic and acetic acids on bacterial growth as optical density at 700 nm.

		Formic acid					Acetic acid				
**Parameters**		**Bs**	**Cb**	**Ec**	**Ln**	**La**	**Bs**	**Cb**	**Ec**	**Ln**	**La**

	*X_m _*(AU)	6.40 ± 0.10	1.15 ± 0.01	2.67 ± 0.04	1.19 ± 0.01	0.79 ± 0.01	6.23 ± 0.13	1.01 ± 0.03	2.87 ± 0.06	0.97 ± 0.03	0.80 ± 0.02
growth model	*v_m _*(AU h^-1^)	0.49 ± 0.03	0.18 ± 0.01	0.70 ± 0.04	0.27 ± 0.01	0.11 ± 0.01	0.51 ± 0.03	0.19 ± 0.02	0.69 ± 0.06	0.12 ± 0.01	0.11 ± 0.06
	*λ *(h)	3.62 ± 0.29	1.81 ± 0.21	0.91 ± 0.16	1.32 ± 0.11	0.94 ± 0.21	3.19 ± 0.40	1.40 ± 0.21	0.52 ± 0.21	0.77 ± 0.28	0.79 ± 0.18

	*K_x_*	0.87 ± 0.11	0.98 ± 0.03	0.81 ± 0.05	1.00 ± 0.01	0.83 ± 0.10	NS	0.37 ± 0.17	0.74 ± 0.24	1.00 ± 0.52	0.87 ± 0.11
effect on *X_m_*	*m_x _*(mM)	35.95 ± 34.50	25.96 ± 1.02	18.23 ± 16.24	29.04 ± 1.30	5.59 ± 1.09	NS	15.78 ± 12.80	48.68 ± 16.29	71.35 ± 55.67	7.09 ± 6.92
	*a_x_*	29.01 (NS)	2.34 ± 0.26	22.14 ± 12.99	1.15 ± 0.07	2.44 ± 0.94	NS	0.99 ± 0.43	2.30 ± 0.40	1.10 ± 0.26	5.60 ± 5.51

	*K_v_*	0.99 ± 0.02	0.99 ± 0.01	1.02 ± 0.15	1.00 ± 0.01	0.98 ± 0.01	NS	0.98 ± 0.02	0.99 ± 0.03	1.00 ± 0.05	1.00 ± 0.02
effect on *v_m_*	*m_v _*(mM)	35.89 ± 35.02	18.91 ± 2.18	18.36 ± 1.26	15.83 ± 1.71	3.80 ± 0.20	NS	16.94 ± 2.15	25.58 ± 2.40	63.46 ± 5.95	5.66 ± 0.28
	*a_v_*	23.92 (NS)	1.47 ± 0.25	2.12 ± 0.27	0.83 ± 0.10	3.11 ± 0.70	NS	1.00 ± 0.12	2.81 ± 0.97	2.47 ± 2.09	3.07 ± 0.87

	*K_λ_*	NS	0.39 ± 0.33	NS	2.40 ± 2.73	NS	NS	NS	NS	NS	NS
effect on *λ*	*m_λ _*(mM)	NS	4.10 ± 2.19	NS	61.10 ± 41.26	NS	NS	NS	NS	NS	NS
	*a_λ_*	NS	1.78 ± 1.09	NS	6.64 ± 5.38	NS	NS	NS	NS	NS	NS

	*p*-value	< 0.001	< 0.001	< 0.001	< 0.001	< 0.001	< 0.001	< 0.001	< 0.001	< 0.001	< 0.001

	*Bf*	1.01	0.92	1.07	1.00	0.92	1.05	1.00	1.00	1.15	1.16

	*Af*	1.18	1.26	1.16	1.19	1.21	1.20	1.13	1.12	1.21	1.38

	R^2^_adj_	0.993	0.997	0.994	0.998	0.994	0.989	0.990	0.986	0.989	0.995

NS: non significant. R^2^_adj_: adjusted coefficient of multiple determination. *p*-value from Fisher's *F *test (α = 0.05). *B_f _*and *A_f _*are the bias and accuracy factor, respectively. Keys for parameter and bacteria details are described in Tables 6 and 7, respectively.

**Figure 2:**
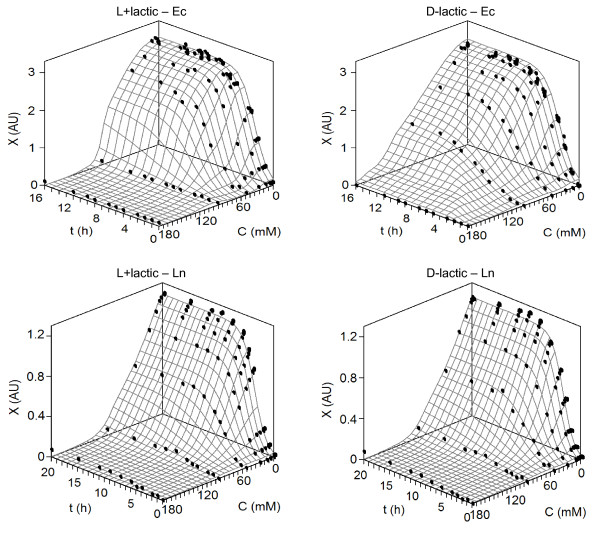
**Experimental data of the growth kinetics for *E. coli *and *L. mesenteroides *affected by D- and L+ lactic acids (points), and fittings to equation (1) (surfaces)**. *X*: growth as optical density at 700 nm (AU); *C*: acid concentration (mM); *t*: time (h). For clarity, confidence intervals (in all cases less than 5% of the experimental mean value; α = 0.05; n = 3) were omitted. Keys for bacteria are described in Table 7.

The numerical results for propionic and butyric acids are shown in Table 2. In all bacterial cultures, these weak acids involved significant changes in two parameters from the logistic equation (maximum bacterial load *X*_m _and maximum growth rate *v*_m_) but not in lag phase (*λ*). However, L+lactic affected the three parameters of growth on Cb and Ec fermentations (Table 3). The lack of significance in the lag phase was clear in the rest of bacteria. In addition, the comparative effect between the two isomeric forms of lactic acid (D- and L+) was also assayed for these last bacteria. Two of these cases, Ec and Ln, are depicted in Figure 2.

**Table 2:** Parametric estimates and confidence intervals (α = 0.05) corresponding to equation (1) applied to the effect of propionic and butyric acids on bacterial growth as optical density at 700 nm.

		Propionic acid					Butyric acid				
**Parameters**		**Bs**	**Cb**	**Ec**	**Ln**	**La**	**Bs**	**Cb**	**Ec**	**Ln**	**La**

	*X_m _*(AU)	6.85 ± 0.12	1.03 ± 0.02	2.78 ± 0.03	1.26 ± 0.01	0.80 ± 0.01	6.35 ± 0.09	0.95 ± 0.02	2.64 ± 0.04	1.13 ± 0.01	0.80 ± 0.02
growth model	*v_m _*(AU h^-1^)	0.49 ± 0.02	0.18 ± 0.01	0.83 ± 0.05	0.29 ± 0.02	0.11 ± 0.01	0.48 ± 0.02	0.17 ± 0.02	0.58 ± 0.04	0.28 ± 0.01	0.10 ± 0.01
	*λ *(h)	3.41 ± 0.31	1.68 ± 0.17	1.02 ± 0.10	1.59 ± 0.12	0.62 ± 0.21	3.19 ± 0.29	0.68 ± 0.24	1.18 ± 0.16	1.33 ± 0.11	0.71 ± 0.28

	*K_x_*	0.91 ± 0.14	0.92 ± 0.15	0.91 ± 0.12	0.99 ± 0.07	0.88 ± 0.12	0.89 ± 0.13	1.00 ± 0.03	1.00 ± 0.05	1.00 ± 0.53	0.97 ± 0.03
effect on *X_m_*	*m_x _*(mM)	33.51 ± 4.99	40.21 ± 11.01	34.79 ± 26.46	70.88 ± 7.07	5.95 ± 5.71	25.51 ± 3.07	45.09 ± 4.43	34.72 ± 23.32	67.57 ± 47.27	6.60 ± 0.72
	*a_x_*	3.45 ± 1.05	1.14 ± 0.22	33.61 ± 9.90	1.43 ± 0.15	6.79 ± 5.94	3.36 ± 0.84	2.60 ± 0.56	16.97 ± 15.12	1.17 ± 0.25	3.17 ± 1.21

	*K_v_*	0.98 ± 0.02	1.00 ± 0.02	1.06 ± 0.09	1.00 ± 0.01	0.99 ± 0.01	0.98 ± 0.02	1.00 ± 0.10	1.03 ± 0.15	1.00 ± 0.02	0.98 ± 0.04
effect on *v_m_*	*m_v _*(mM)	39.46 ± 15.0	17.35 ± 1.73	21.86 ± 1.87	39.72 ± 3.19	4.80 ± 0.38	34.09 ± 32.69	20.00 ± 3.42	23.56 ± 3.21	28.05 ± 2.25	4.35 ± 0.39
	*a_v_*	17.34 ± 8.99	0.99 ± 0.11	2.03 ± 0.51	1.08 ± 0.10	3.72 ± 2.03	22.91 ± 20.94	1.20 ± 0.25	2.23 ± 0.34	1.12 ± 0.11	1.97 ± 0.35

	*K_λ_*	NS	NS	NS	NS	NS	NS	NS	NS	NS	NS
effect on *λ*	*m_λ _*(mM)	NS	NS	NS	NS	NS	NS	NS	NS	NS	NS
	*a_λ_*	NS	NS	NS	NS	NS	NS	NS	NS	NS	NS

	*p*-value	< 0.001	< 0.001	< 0.001	< 0.001	< 0.001	< 0.001	< 0.001	< 0.001	< 0.001	< 0.001

	*Bf*	1.03	1.06	1.11	1.02	1.08	1.02	1.14	1.11	1.05	1.08

	*Af*	1.19	1.18	1.19	1.06	1.16	1.22	1.21	1.18	1.12	1.36

	R^2^_adj_	0.992	0.995	0.995	0.997	0.993	0.992	0.988	0.993	0.996	0.988

NS: non significant. R^2^_adj_: adjusted coefficient of multiple determination. *p*-value from Fisher's *F *test (α = 0.05). *B_f _*and *A_f _*are the bias and accuracy factor, respectively. Keys for parameter and bacteria details are described in Tables 6 and 7, respectively.

**Table 3:** Parametric estimates and confidence intervals (α = 0.05) corresponding to equation (1) applied to the effect of lactic acids (two isomeric forms) on bacterial growth as optical density at 700 nm.

		L+Lactic acid					D-Lactic acid		
**Parameters**		**Bs**	**Cb**	**Ec**	**Ln**	**La**	**Cb**	**Ec**	**Ln**

	*X_m _*(AU)	7.19 ± 0.21	0.88 ± 0.02	2.80 ± 0.04	1.18 ± 0.01	0.78 ± 0.01	0.85 ± 0.02	2.82 ± 0.04	1.16 ± 0.02
growth model	*v_m _*(AU h^-1^)	0.47 ± 0.04	0.17 ± 0.01	0.80 ± 0.06	0.30 ± 0.01	0.11 ± 0.01	0.19 ± 0.02	0.73 ± 0.05	0.29 ± 0.02
	*λ *(h)	4.48 ± 0.55	1.14 ± 0.18	0.74 ± 0.15	1.51 ± 0.15	1.09 ± 0.18	1.18 ± 0.18	0.85 ± 0.15	1.35 ± 0.12

	*K_x_*	0.88 ± 0.24	0.81 ± 0.20	1.00 ± 0.04	0.72 ± 0.14	0.94 ± 0.05	0.98 ± 0.29	1.00 ± 0.05	0.94 ± 0.22
effect on *X_m_*	*m_x _*(mM)	44.48 ± 19.79	20.17 ± 7.08	63.17 ± 12.47	31.22 ± 7.96	4.58 ± 4.15	23.22 ± 11.20	69.28 ± 6.28	40.25 ± 13.34
	*a_x_*	32.00 ± 25.73	1.15 ± 0.22	3.00 ± 0.73	1.38 ± 0.16	5.82 ± 5.01	0.88 ± 0.19	1.70 ± 0.28	1.21 ± 0.19

	*K_v_*	NS	1.00 ± 0.03	1.00 ± 0.04	1.00 ± 0.02	1.00 ± 0.01	1.00 ± 0.03	0.82 ± 0.07	0.99 ± 0.02
effect on *v_m_*	*m_v _*(mM)	NS	17.18 ± 2.95	51.07 ± 21.89	29.23 ± 3.88	5.00 ± 0.70	15.80 ± 2.48	36.47 ± 5.80	25.14 ± 2.54
	*a_v_*	NS	1.04 ± 0.19	4.00 ± 4.21	1.50 ± 0.35	2.02 ± 0.71	1.31 ± 0.24	1.91 ± 1.53	1.42 ± 0.19

	*K_λ_*	NS	11.89 ± 11.70	2.52 ± 1.15	NS	NS	NS	0.88 ± 0.85	NS
effect on *λ*	*m_λ _*(mM)	NS	50.80 ± 20.16	18.98 ± 4.86	NS	NS	NS	19.09 ± 11.30	NS
	*a_λ_*	NS	4.58 ± 3.62	2.82 ± 1.47	NS	NS	NS	3.25 ± 3.40	NS

	*p*-value	< 0.001	< 0.001	< 0.001	< 0.001	< 0.001	< 0.001	< 0.001	< 0.001

	*Bf*	1.12	1.15	1.00	1.07	0.88	1.09	1.10	1.10

	*Af*	1.32	1.16	1.09	1.12	1.18	1.26	1.15	1.14

	R^2^_adj_	0.954	0.993	0.991	0.996	0.995	0.989	0.992	0.994

NS: non significant. R^2^_adj_: adjusted coefficient of multiple determination. *p*-value from Fisher's *F *test (α = 0.05). *B_f _*and *A_f _*are the bias and accuracy factor, respectively. Keys for parameter and bacteria details are described in Tables 6 and 7, respectively.

The fitting of results was always satisfactory both graphically and statistically. The mathematical equations were robust and consistent (*p*-values < 0.001 from Fisher's *F *test), the residuals were randomly distributed and autocorrelations were not observed by Durbin-Watson test (data not shown). Furthermore, all the adjusted coefficients of multiple determination between predicted and observed values were always R^2^_adj _> 0.95, with a wide majority of the fittings superior at 0.99. Bias and accuracy factors (*Bf *and *Af*) also indicated the lack of bias and high accuracy of equation (1) to describe experimental effects of acids on bacterial growth.

For the global description of the carboxylic acid toxicity, a single index -*EC_50,τ _*- was used (Table 4). This parameter is a summary of all the effects on the biomass produced at a given time -τ- or time required to achieve the semi-maximum biomass [22]. This value could be the main parameter with practical interest in terms of environmental assessment and operational applications of chemicals. Thereby, comparison of toxicity among different agents and bacterial sensitivity can be also evaluated using this parameter. Our results revealed that the toxicity of carboxylic acids almost always decreased (higher value of *EC_50,τ_*) with increasing of their molecular weights but not depending on pK_a _value. Thus, formic and acetic acids led to greater toxic values than butyric and lactic acids. In all treatments, La was the microorganism most susceptible to the acids effects and Ln and Bs the most resistant. In addition, L+lactic was more toxic than D-lactic on Ec growth whereas the effect of D-lactic on Ln was higher than its optical isomer.

**Table 4:** Values of *EC_50,τ; _*(mM) describing the effect of carboxylic acids on bacterial growth.

	Bacteria				
	
Carboxylic acids	Bs	Cb	Ec	Ln	La
Formic	35.21	14.65	16.91	17.47	3.52
Acetic	31.60	18.29	25.84	61.25	5.20
Propionic	23.23	16.07	23.42	39.06	5.09
Butyric	31.90	17.12	28.73	31.58	3.96
L+lactic	44.42	17.13	21.36	28.97	4.51
D-lactic	-	18.47	31.51	25.84	-

Keys for bacteria are described in Table 7.

**Figure 3:**
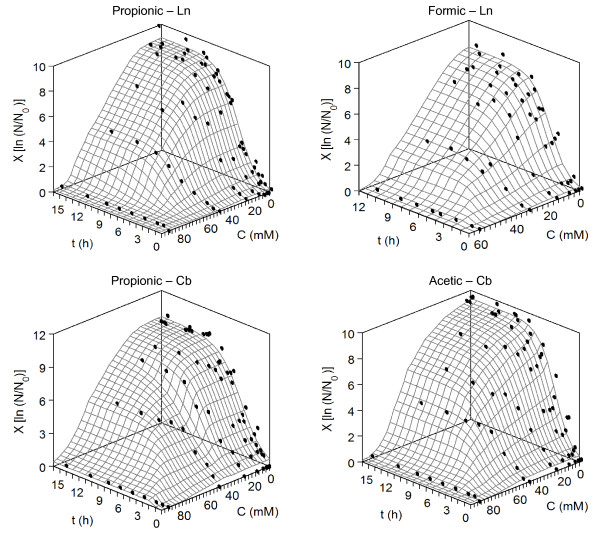
**Growth data as relative cell viable counts for *C. piscicola *and *L. mesenteroides *affected by different acids (points)**. Response surfaces show growth predicted by equation (1). *X*: growth quantified by ln (N/N_0_); *C*: acid concentration (mM); *t*: time (h). For clarity, confidence intervals (in all cases less than 5% of the experimental mean value; α = 0.05; n = 3) were omitted. Keys for bacteria are described in Table 7.

The validation and generalization of equation (1) was studied using relative viable cell count as dependent variable of bacterial load. Four cases were selected and mathematical description was elaborated (Figure 3). The parameter estimations were in agreement with previously reported using optical density at 700 nm (Table 5). Lag phase of Cb and Ln was not significantly affected by acetic, formic and propionic acids. However, both parameters, *X_m _*and *v_m_*, were significantly modified by those acids. The statistical consistency, goodness-of-fit, lack of autocorrelations for residuals and high predictability of the model was clearly showed.

**Table 5:** Parametric estimations and confidence intervals (α = 0.05) corresponding to the equation (1) applied to the selected acids on bacterial growth measured by relative viable cell count.

Parameters		Acetic_Cb	Propionic_Cb	Formic_Ln	Propionic_Ln
	*X_m_*	8.84 ± 0.44	9.78 ± 0.39	8.33 ± 0.36	8.58 ± 0.30
growth model	*v_m _*(h^-1^)	1.79 ± 0.36	1.52 ± 0.12	1.57 ± 0.38	1.81 ± 0.12
	*λ *(h)	0.82 ± 0.40	0.93 ± 0.91	0.89 ± 0.40	2.81 ± 2.00

	*K_x_*	1.00 ± 0.09	1.00 ± 0.52	1.00 ± 0.06	0.67 ± 0.28
effect on *X_m_*	*m_x _*(mM)	25.21 ± 3.91	32.18 ± 16.86	19.39 ± 2.26	19.81 ± 8.78
	*a_x_*	1.35 ± 0.29	1.53 ± 0.59	1.66 ± 0.56	2.76 ± 0.51

	*K_v_*	1.35 ± 0.96	0.99 ± 0.11	0.69 ± 0.67	1.00 ± 0.95
effect on *v_m_*	*m_v _*(mM)	14.91 ± 12.19	40.34 ± 12.64	14.67 ± 10.12	36.67 ± 27.97
	*a_v_*	0.56 ± 0.42	0.64 ± 0.31	1.90 ± 1.06	5.32 ± 2.31

	*K_λ_*	NS	NS	NS	NS
effect on *λ*	*m_λ _*(mM)	NS	NS	NS	NS
	*a_λ_*	NS	NS	NS	NS

	*EC_50,τ_*	14.99	29.19	15.74	33.77

	*p*-value	< 0.001	< 0.001	< 0.001	< 0.001

	*Bf*	1.15	0.98	1.16	0.88

	*Af*	1.30	1.15	1.23	1.34

	R^2^_adj_	0.974	0.970	0.975	0.977

*EC_50,__τ_*is expressed in mM. NS: non significant. R^2^_adj_: adjusted coefficient of multiple determination. *p*-value from Fisher's *F *test (α = 0.05). Keys for parameter and bacteria details are described in Tables 6 and 7, respectively.

## Discussion

The combination of sigmoid equations for modelling DR relationships of agents affecting the most important parameters from microbial growth has been scarcely reported. However, excellent results were obtained when it was applied to the study of detergent [20] and heavy metals [22] on algae and bacterial kinetics, respectively. From the results of acid-dosed cultures performed in this study, it can be seen that the bacterial system showed different profiles of growth and responses to the toxic effect of carboxylic acids. All 3D-surfaces of experimental data were perfectly modelled by the proposed equation (1) and good predictability of this bivariate model was also demonstrated. Statistical analysis confirmed these results. In all cases, distribution of residuals was randomly scattered around zero and grouped data and autocorrelations were not observed. Adjusted coefficients of multiple determination indicated the goodness of fit and *p*-values from Fisher's *F *test showed the robustness and consistence of mathematical model. *Bf *and *Af *factors also revealed the good agreement between experimental and simulated data. In addition, we have shown that the parameter -*EC_50,τ _*- used in the global characterization of toxic effects by carboxylic acids produces an interesting resource to compare toxicities between chemicals and sensibility of microorganisms to them.

Although absorbance measurements have been less used within microbiological modelling, it is a simple and inexpensive technique, in comparison with laborious and expensive plate count methodology, for generation of several growth data (*i.e*., bivariate experiments) that allow the formulation of accuracy mathematical models as equation (1). This equation was also validated using relative population size [24] as growth response. With present data, the use of relative viable cell count as dependent variable avoided the problem reported by Dalgaard and Koutsoumanis [25], in where the differences of lag phase and rate parameters obtained by two kinds of bacterial load quantification (count plate and absorbance) limited the ability of comparison among different experimental conditions. Indeed, the units of parameters and the numerical value of them are different (Tables 6 and 5) but behaviour among two approaches was easily comparable.

**Table 6:** Symbolic notations used and corresponding units.

*Growth dynamics measured by optical density*	
*X*:	Growth measured as absorbance at 700 nm. Units: absorbance units (AU)
*t*:	Time. Units: h
*X_m _*:	Maximum bacterial load. Units: absorbance units (AU)
*v_m _*:	Maximum growth rate. Units: AU h^-1^
*λ*	Lag phase. Units: h
*X_m•_*:	Maximum bacterial load affected by chemical agent. Units: absorbance units (AU)
*v_m•_*:	Maximum growth rate affected by chemical agent. Units: AU h^-1^
*λ*_•_:	Lag phase affected by chemical agent. Units: h

***Growth dynamics measured by viable cell counts***	

*X*:	Growth as relative cell viable count or relative population size [ln (N/N_0_)]. Units: Dimensionless
*t*:	Time. Units: h
*X_m_*:	Maximum bacterial load. Units: Dimensionless
*v_m_*:	Maximum specific growth rate. Units: h^-1^
*λ*	Lag phase. Units: h
*X_m•_*:	Maximum bacterial load affected by chemical agent. Units: Dimensionless
*v_m•_*:	Maximum specific growth rate affected by chemical agent. Units: h^-1^
*λ*_•_:	Lag phase affected by chemical agent. Units: h

***Dose effects on growth dynamic ***	

*C*:	Concentration of chemical agent (acid). Units: mM
*K_x_*:	Maximum response affecting on *X_m_*. Dimensionless
*m_x_*:	Concentration corresponding to the semi-maximum response affecting on *X_m_*. Units: mM
*a_x_*:	Shape parameter affecting on *X_m_*. Dimensionless
*K_v_*:	Maximum response affecting on *v_m_*. Dimensionless
*m_v_*:	Concentration corresponding to the semi-maximum response affecting on *v_m_*. Units: mM
*a_v_*:	Shape parameter affecting on *v_m_*. Dimensionless
*K_λ_*:	Maximum response affecting on *λ*. Dimensionless
*m_λ_*:	Concentration corresponding to the semi-maximum response affecting on *λ*. Units: mM
*a_λ_*:	Shape parameter affecting on *λ*. Dimensionless

In the cell measurement, the parameter of rate from equation (1) was defined as maximum specific growth rate (units of h^-1^) meanwhile, in optical quantification, it was denominated as maximum growth rate (units of AU h^-1^). Both parameters are related in the description of the growth rate of bacteria but those estimates can not be directly compared. However, the tendencies predicted by proposed equation in both quantifications of growth were similar and statistically significant.

Previous works have reported the importance of the concentration of carboxylic acid exposures in suppressing the growth of bacteria [7,26-28]. Conventional studies of bacterial resistance [29], adaptation [9] and membrane permeabilization [30] have demonstrated the dependence of metabolic responses with the microorganism and acid concentration evaluated. Nonetheless, these results have not been used optimally to characterize mechanisms and modes of action underlying the effects, to guide the selection of dosing ranges and to compare the effectiveness of acid treatments. These descriptions are very important when weak acids must be applied to reduce the levels of food-borne pathogens on food processing, control industrial fermentations or avoid contaminations on surfaces and devices. Consequently, our modelling approach provides a simple and consistent tool for such descriptions.

In all cases, we have found that carboxylic acids significantly affected the kinetic parameters, maximum biomass and maximum growth rate, but only in specific cultures the lag phase was altered by these chemicals. These heterogeneous responses in the lag phase duration have been also observed using heavy metals [22,31], lactic acid [32] and salts and bacteriocins [33] as effectors. Cherrington et al. [34] suggested that direct comparisons of different organic acids in relation to their antimicrobial activities are difficult due to the variation in physical characteristics. However, we have demonstrated that the effects and toxicity of carboxylic acids can be successfully compared and significant kinetic parameters can be obtained. Thus, the toxicity was associated to the molecular weight and the sensitivity of bacteria was very varied being La the most clearly sensitive to acids (Table 4). Regarding with stereoisomers of lactic acid, antimicrobial capacity of D-lactic on Cb and Ec fermentations was lower than L+lactic. This finding is in agreement with previously reported by Leitch and Stewart [27] in which the susceptibility of several strains of *E. coli *was greater to L+lactic than that observed with D-lactic, but not depending on transmembrane pH gradient.

## Conclusions

In summary, a general bivariate model has been proposed for the characterization of the effects from five carboxylic acids (formic, acetic propionic, butyric and lactic) against five bacteria (*C. piscicola*, *E. coli*, *B. subtilis*, *L. mesenteroides *and *L. anguillarum*). This equation could be easily applied to other chemicals and microorganisms and may support a resource for exhaustive comparison and formal description of agent effects on bacterial growth. Toxicity of acids decreased with the increase of molecular weight and *L. anguillarum *was the least resistant bacterium. In addition, L+lactic was more toxic than D-lactic for the growth of *C. piscicola *and *E. coli*.

## Methods

### Microbiological methods and chemicals

The studied bacteria are shown in Table 7. These microorganisms were chosen by their different features about cell wall structure (Gram-positive and negative), behaviour (free, opportunistic parasite, probiotic), habitats (marine and terrestrial) and metabolic properties (homo and heterofermentative). *L. anguillarum *and *L. mesenteroides *were kindly provided by Dr. Harry Birkbeck (University of Glasgow, UK) and Dr. B. Ray (University of Wyoming, Laramie, USA), respectively.

**Table 7:** Bacteria used.

Bacteria	Strain	Key
*Bacillus subtilis*	CECT 35	Bs
*Carnobacterium piscicola*	CECT 4020	Cb
*Escherichia coli*	CECT 731	Ec
*Leuconostoc mesenteroides *subsp. *lysis*	HD-IIM_1	Ln
*Listonella anguillarum*	90-11-287*	La

CECT: Spanish Type Culture Collection (University of Valencia, Spain).

HD-IIM: Department Animal Science, University of Wyoming (Wyoming, USA)

*Listonella anguillarum was isolated from rainbow trout and initially defined as *Vibrio anguillarum *[35].

Stock cultures of bacteria were kept at -80°C in commercial MRS (*L. mesenteroides *HD-IIM_1 and *C. piscicola *CECT 4020), marine (*L. anguillarum *90-11-287) and tryptone soy broths (*B. subtilis *CECT 35 and *E. coli *CECT 731) with 25% glycerol [36,37]. Marine medium were provided by Difco (Becton, Dickinson and Company, MD, USA), MRS (de Man, Rogosa and Sharpe) medium by Pronadisa (Hispanlab S.A., Spain) and tryptone soy broth (TSB) by Panreac (Panreac Química SA, Barcelona, Spain). Culture media were prepared as indicating on commercial formulation and sterilized at 121°C for 15 min. Carboxylic acids were in all cases purchased from Sigma-Aldrich (St. Louis, MO, USA). Concentrated solutions of these acids were separately prepared and sterilized with steam flow at 101°C for 1 h.

### Bioassay and culture conditions

Fermentations were carried out in triplicate using methods which were described in detail in previous reports [38,39]. To prepare the microbial suspensions, 12-h cultures in commercial media were centrifuged at 4,000 g for 15 min and the sediments washed with phosphate buffer 0.005 M. Subsequently, the sediments were again centrifuged and resuspended in the corresponding buffed medium for each bacterium: 0.05 M pH = 6.0 biphtalate-NaOH buffer in MRS (Ln and Cb), 0.05 M pH = 7.4 Tris-HCl buffer in marine medium (La) and 0.05 M pH = 7.4 Tris-HCl buffer in TSB (Bs and Ec) and adjusted to an absorbance (700 nm, A_700_) of 0.200. For bioassay, equal volumes of individual concentrations of each acid (Table 8) and microbial suspension were mixed in 15 ml tubes. Thus, under these conditions the inocula concentrations for the tested bacteria were established among 10^6 ^and 10^7 ^cfu ml^-1^. All incubations were performed with orbital shaking at 200 rpm and 22°C (La), 30°C (Ln, Cb) and 37°C (Bs, Ec).

**Table 8:** Range of final acid concentrations (mM) used in each culture.

	Bacteria				
	
Carboxylic acids	Bs	Cb	Ec	Ln	La
Formic	272-(:2)-0	543-(:2)-0	272-(:2)-0	543-(:2)-0	109-(:2)-0
Acetic	266-(:2)-0	266-(:2)-0	266-(:2)-0	266-(:2)-0	83-(:2)-0
Propionic	169-(:2)-0	338-(:2)-0	169-(:2)-0	675-(:2)-0	68-(:2)-0
Butyric	142-(:2)-0	142-(:2)-0	142-(:2)-0	142-(:2)-0	57-(:2)-0
L+lactic	178-(:2)-0	178-(:2)-0	178-(:2)-0	178-(:2)-0	56-(:2)-0
D-lactic	-	178-(:2)-0	178-(:2)-0	178-(:2)-0	-

The concentrations tested in each case were formulated from the first value of the range and 9 serial twofold dilutions (:2) as well as a control without carboxylic acid. Keys for bacteria are described in Table 7.

At pre-established times, samples were centrifuged at 4,000 g for 15 min. Sediments (bacterial biomass) were washed and resuspended in distilled water to the appropriate dilution for measuring the bacterial growth by A_700_. For comparative purposes, some sediments were used for quantify viable cells by means of plate count technique on commercial media with agar. Serial, 10-fold dilutions were prepared in peptone-buffered solutions and 0.1 ml samples were plated in triplicate, incubated at previously indicated temperatures and manually counted (after 3 days of incubation). Results were expressed as relative viable cell count [24,37]: ln (N/N_0_); where N is the colony-forming units per ml (cfu ml^-1^) and N_0 _is the initial cfu ml^-1^.

### Mathematical modelling

#### Dose-growth model

Recently, we have proposed a bivariate equation (secondary model) that modelled satisfactorily the effect of three heavy metals on the growth parameters of several bacteria [22]. This equation is based on the combination of Weibull function as DR model [40,41] affecting the most important parameters of the reparametrized logistic equation used for growth description [42]:

(1) $$X=\frac{X_{m∙}}{1+\exp\left[2+\frac{4v_{m∙}}{X_{m∙}}\left(\lambda_{∙}-t\right)\right]}\;;\;\text{where:}$$

$$X_{m∙}=X_{m}\left\{1-K_{x}\left[1-\exp\left(-\ln2{\left(C∕m_{x}\right)}^{a_{x}}\right)\right]\right\}$$

$$v_{m∙}=v_{m}\left\{1-K_{v}\left[1-\exp\left(-\ln2{\left(C∕m_{v}\right)}^{a_{v}}\right)\right]\right\}$$

$$\lambda_{∙}=\lambda \left\{1+K_{\lambda }\left[1-\exp\left(-\ln2{\left(C∕m_{\lambda }\right)}^{a_{\lambda }}\right)\right]\right\}$$

This equation can be easily and accurately used when the dependent variable or response (growth and population size) is described by means of optical density (absorbance at 700 nm) or relative viable cell count (ln (N/N_0_)). In the present work, *X *is the growth measured as optical density or relative population size, *v_m _*is the maximum growth rate, *X_m _*is the maximum bacterial load (growth), *λ *is the lag phase and *C *is the acid concentration. The meaning of the rest of symbolic notations and corresponding units are summarized in Table 6.

In addition, a global parameter (*EC_50,τ_*) was also defined for the overall description of chemical effects on kinetic studies. This parameter was defined as the dose of agent (in mM) that reduces the biomass by 50% compared to that produced by the control at time (*τ*) that reduces the biomass by 50% [22].

### Numerical methods

Fitting procedures and parametric estimates from the experimental results were performed by minimisation of the sum of quadratic differences between observed and model-predicted values, using the nonlinear least-squares (quasi-Newton) method provided by the macro '*Solver*' of *Microsoft Excel *2003 spreadsheet. Firstly, growth curves without acid were fitted and subsequently effect of acid on parameters was calculated with fixed values of *X_m_*, *v_m _*and *λ*. For relative viable cell count, an assumption was established: when (N/N_0_) data were < 1 then values of ln (N/N_0_) were forced to zero. Subsequently, confidence intervals from the parametric estimates (Student's *t *test), consistence of mathematical models (Fisher's *F *test) and residual analysis (Durbin-Watson test) were determined with '*SolverAid*' macro, which is freely available from de Levie's Excellaneous website: http://www.bowdoin.edu/~rdelevie/excellaneous/. In addition, bias and accuracy factors of the equation (1) were calculated to evaluate the fitting of that model to experimental data [43]:

(2) $$Bf=10^{\frac{\sum \log\left(\frac{predicted}{observed}\right)}{n}}$$

(3) $$Af=10^{\frac{\sum \left|\log\left(\frac{predicted}{observed}\right)\right|}{n}}$$

where log (predicted/observed) is the logarithmic relation between the predicted and the experimental values, and *n *is the number of data. The nearer the values of *Bf *(bias factor) and *Af *(accuracy factor) to 1 indicate the better the fitting of the models to experimental data (a value of 1 indicates that there is perfect agreement among predicted and observed data).

## Competing interests

The authors declare that they have no competing interests.

## Authors' contributions

JAV performed the experiments, worked on mathematical modelling of experimental data and wrote the manuscript. AD, IRA, MAP and DR helped in the analytical determinations. MAM developed the mathematical models and designed the present study. All authors read and approved the manuscript.
